# A Statistical-Textural-Features Based Approach for Classification of Solid Drugs Using Surface Microscopic Images

**DOI:** 10.1155/2014/791246

**Published:** 2014-10-13

**Authors:** Fahima Tahir, Muhammad Abuzar Fahiem

**Affiliations:** Department of Computer Science, Lahore College for Women University, Jail Road, Lahore 54000, Pakistan

## Abstract

The quality of pharmaceutical products plays an important role in pharmaceutical industry as well as in our lives. Usage of defective tablets can be harmful for patients. In this research we proposed a nondestructive method to identify defective and nondefective tablets using their surface morphology. Three different environmental factors temperature, humidity and moisture are analyzed to evaluate the performance of the proposed method. Multiple textural features are extracted from the surface of the defective and nondefective tablets. These textural features are gray level cooccurrence matrix, run length matrix, histogram, autoregressive model and HAAR wavelet. Total textural features extracted from images are 281. We performed an analysis on all those 281, top 15, and top 2 features. Top 15 features are extracted using three different feature reduction techniques: chi-square, gain ratio and relief-F. In this research we have used three different classifiers: support vector machine, *K*-nearest neighbors and naïve Bayes to calculate the accuracies against proposed method using two experiments, that is, leave-one-out cross-validation technique and train test models. We tested each classifier against all selected features and then performed the comparison of their results. The experimental work resulted in that in most of the cases SVM performed better than the other two classifiers.

## 1. Introduction

Pharmaceutical drugs are chemical compounds that can be used to preclude and cure patients from different kinds of diseases. In today's fast moving era, the advancements in the field of pharmacology help doctors to save lives of people by curing them. Tablets are the most common form of medicines prescribed by physicians to the patients. U.S. FDA (Food and Drug Administration) is responsible for approving the medicines before sending to the local market by their manufacturers. FDA allows only those medicines to sell in the market that are safe and fulfil all the quality metrics defined by them.

When these medicines are supplied to the local pharmacies even after approval by the FDA there are still many chances that the medicines are substandard. Substandard medicines are those that somehow do not fulfill the quality standards and are harmful for the patient's health. They can be categorized as counterfeit, expired and environment-affected medicines.

Environment-affected medicines are those which conform to the standards at the time of manufacturing but with the passage of time different external factors change them into the category of substandard medicines. These factors include moisture, light (especially sunlight), extreme temperature, and oxygen. As discussed by Islam et al. [1], moisture affects the physical and chemical stability of the drugs by accelerating the hydrolysis and reacting with the excipients. In another research Szakonyi and Zelkó states [2] water absorption in the surface of a tablet results in degradation of its active pharmaceutical ingredients (APIs). The use of defective tablets may cause some minor issues in the patient's body like allergies or may result in their death. So there is an immense need of such a method that can identify environmental affected medicines after their manufacturing.

In this research we are dealing with three environmental factors, that is, humidity, moisture, and temperature. Humidity means the amount of water vapor available in the air. The APIs of the pharmaceutical tablets indicate reaction with humidity if they left in open air which results in oxidation and reduction processes. The second factor we are dealing in this research is moisture. The term moisture is related to the contents of water in liquid state. The stability of the tablets strongly depends on the amount of water present in them. The increase in the amount of moisture above its actual need can cause reactions of APIs and excipients as discussed in [1]. Similarly temperature is the third environmental factor dealt with in this study. Temperature changes the potency of tablets and results in unpredictable behavior.

Different techniques are available in literature for the assessment and estimation of formulation, quality, correctness, and stability of the solid drugs. Some of these techniques are used at the time of manufacturing to get information about the correct amount of APIs. TLC (thin layer chromatography) and HPLC (high-performance liquid chromatography) are traditional techniques that are used for this purpose. Deisingh [3] uses TLC for the estimation and identification of counterfeit medicines or the APIs from the tablets. Both of these techniques are slow, expensive, and destructive [4].

As discussed in some other researches [3, 5–8], solid drug assessment techniques can also be categorized as spectrum based assessment (SBA) techniques. These include mass spectrometry (MS), nuclear magnetic resonance spectroscopy (NMR), X-ray diffraction (XRD), scanning electron microscopy (SEM), and vibrational spectroscopic (VS) techniques. VS include Raman and near-infrared spectroscopy techniques. Different researches [9–11] explain that all of these require either full or some amount of sample preparation so they are either destructive or semidestructive except that of the VS technique.

Spectral image based assessment (SIBA) techniques are another type that can be used for the analysis of solid form of dosages. SIBA involves two major techniques known as multispectral imaging (MSI) and hyperspectral imaging (HSI). Hamilton and Lodder [12] use HSI for the analysis of pharmaceutical medicines to compare the performance of HSI over HPLC and conclude that HSI is more accurate. In another research, Gowen et al. [13] performed nondestructive assessment of the pharmaceutical tablets using VS along with various image processing (IP) techniques. The image created from the combination of digital imaging with either Raman spectroscopy or near-infrared spectroscopy are known as chemical image. Chemical imaging is used by Šašić [14] for the analysis of pharmaceutical raw ingredients. From different researches [15–17] it is found that chemical imaging can also be used to monitor the development process and quality control of the pharmaceutical tablets. Puchert et al. [18] uses near-infrared chemical imaging (NIRCI) for the identification of counterfeit medicines. Extensive comparative studies of all these techniques are available in [5, 19].

Image based assessment (IBA) is also used for the analysis and classification of the tablets. IBA is a nondestructive, less expensive, and simple approach based on different IP techniques like image enhancement, segmentation, edge, contour detection and texture analysis, and so forth. Segmentation of grayscale tablet images using adaptive thresholding and morphological operations is used for the tablet identification which is also known as pill recognition. Andreas et al. in his researches [20, 21] performed classification using Euclidean distance on a feature set based on size, shape, and color, and the results describe that the most dominant feature from these three is “size.” Ramya et al. [22] used template matching along with a series of IP techniques to detect broken tablets from blister packaging. Špiclin et al. [23] performed inspection of imprinted tablets using image registration on an image database of different defective and nondefective tablets. They used three registration methods in this research: direct matching of pixel intensities, principal axis matching, and circular profile matching. Comparative analysis shows that circular profile matching is a more powerful registration technique of visual inspection of the tablets. In another research comparison of geometrical and statistical methods for visual inspection of tablets was performed using receiver operating characteristics analysis. Geometrical features are based on imprinted shape while on the other hand statistical features are based on tablet surface statistics. The proposed inspection method by Bukovec [24] can identity five types of defects that are spot, deboss, emboss, crack, and dot. Results show that the features extracted from the statistical methods are better than the geometrical methods for the tablet inspection.

In this research we are focusing on the IBA of the tablet surface morphology using textural features. The proposed methodology helps in classification of the solid tablets into two different categories, defective tablets (DT) and nondefective tablets (NDT). The research aims at formulating a new nondestructive method based on the surface analysis of tablets for their abovesaid classification. In the rest of the paper Section 2 provides the material and methods, Section 3 describes results and discussions, and Section 4 concludes the paper.

## 2. Material and Methods

### 2.1. Image Acquisition

To perform the experimentation of the proposed methodology nine different datasets are created. Each dataset comprises the images of defective and nondefective versions of ten different tablets. These images are captured using Labomed 5 MP digital camera mounted on Nikon Eclipse LV100 microscope with a resolution of 2580 × 1944. We considered three major environmental factors, that is, temperature, moisture, and humidity, for the creation of defective tablets.

Three datasets are created for the tablets affected by temperature and labeled as T1, T2, and T3. T1 consists of images of the tablets which are placed in an area having 200°C temperature for five minutes and their nondefective versions. Similarly T2 and T3 contain images of defective and nondefective tablets placed in 240°C and 280°C for five minutes, respectively. In the same way three datasets are created for humidity factor labeled as A1, A2, and A3. Defective tablets in A1 are placed out of their packaging (in open air) for three days; similarly, A2 and A3 contain images of the tablets that remain out of their packaging for two days and one day, respectively. Another three datasets are created for the tablet images affected by moisture. Moisture affected tablet images were captured after affecting four tablets at day 1, four at day 2, and four at day 3 by different levels of moisture (liquid water) which they were exposed to and these datasets of the tablets are referred to as W1, W2, and W3, respectively. A brief description of datasets is given in Table 1.

**Figure 1:**
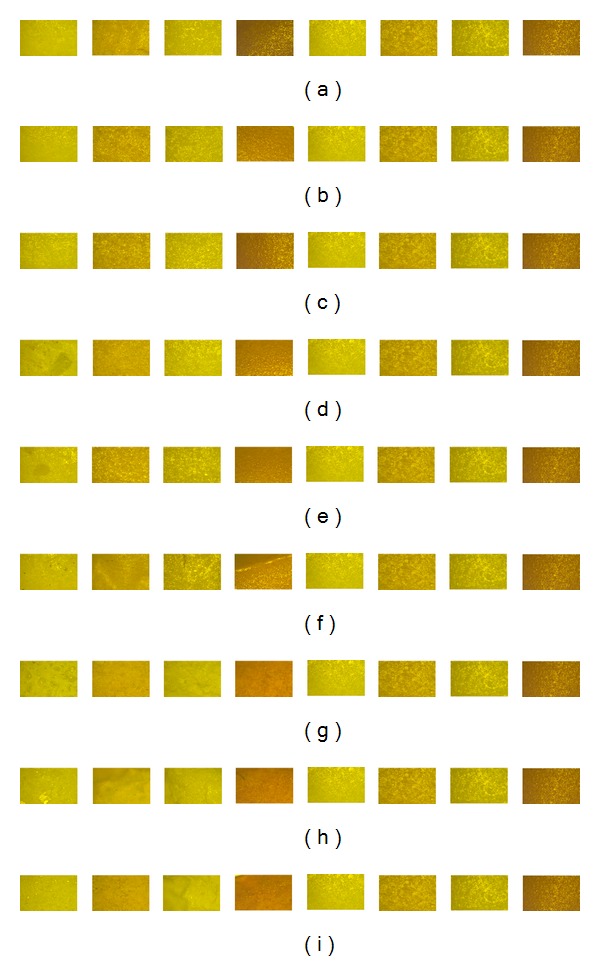
(a) Tablet images contained in dataset A1. (b) Tablet images contained in dataset A2. (c) Tablet images contained in dataset A3. (d) Tablet images contained in dataset T1. (e) Tablet images contained in dataset T2. (f) Tablet images contained in dataset T3. (g) Tablet images contained in dataset W1. (h) Tablet images contained in dataset W2. (i) Tablet images contained in dataset W3.

Figure 1 shows some of the images of the datasets used in this research. In each part of Figure 1 the first four images are of environment-affected tablets and the last four are of their nondefective versions. Figures 1(a), 1(b), and 1(c) parts show tablet images of datasets A1, A2, and A3 which are affected by humidity. Similarly Figures 1(d), 1(e), and 1(f) display tablets affected by temperature and labeled as T1, T2, and T3. Figures 1(g), 1(h) and 1(i) represent tablets of datasets W1, W2, and W3, respectively. Each of these three datasets belongs to moisture affected tablets.

### 2.2. Proposed Methodology

**Figure 2:**
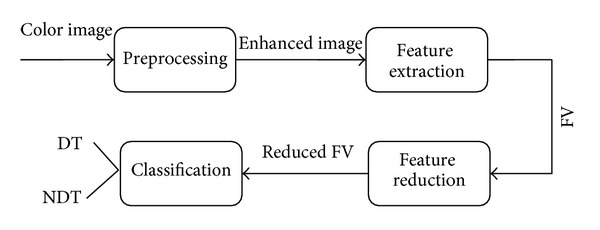
Basic flow of the proposed methodology.

In this research our main focus is on analysis based on surface morphology of the solid dosage forms (tablets) using IP and ML (machine learning). Surface of a tablet can effectively represent its characteristics. In proposed methodology we are using tablet surface images for the classification between DT and NDT. The proposed methodology mainly consists of four phases: preprocessing, feature extraction, feature reduction, and classification. The main flow of the proposed approach is shown in Figure 2.

**Figure 3:**
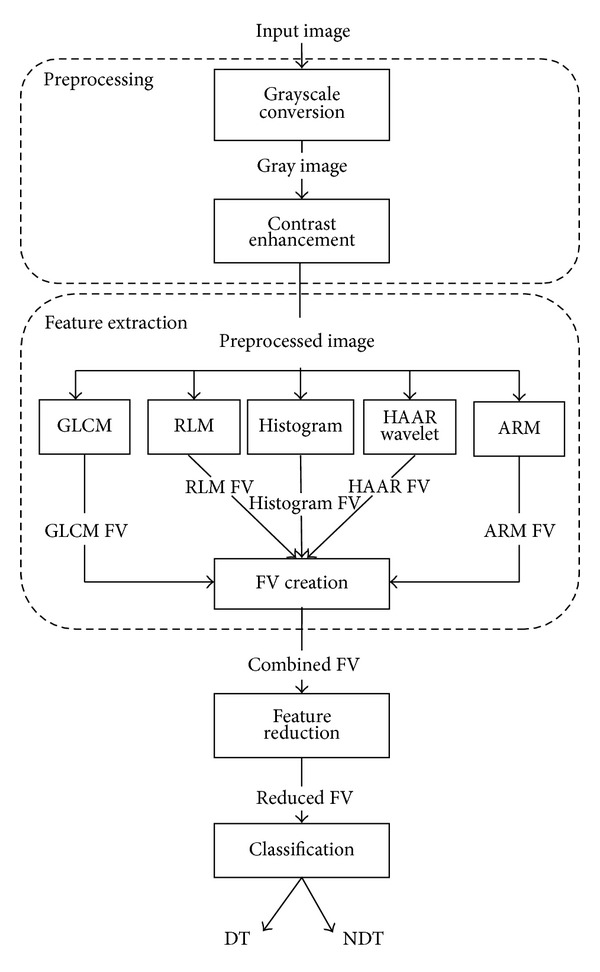
Detailed diagram of the proposed methodology.

In the first phase, input images are prepared for further analysis. The preprocessed images are then passed to feature extraction phase to extract different textural features which will be stored as feature vector (FV). In the next phase, feature reduction techniques are applied on the FV to reduce its dimensionality. The last phase classifies the images into DT and NDT based on the selected features. Details of the proposed methodology are shown in Figure 3.

#### 2.2.1. Preprocessing

Preprocessing consists of algorithms that can be used for image enhancement and noise removal. After image acquisition, preprocessing is an essential step to prepare the captured images for the feature extraction. Preprocessing is performed in two steps, that is, grayscale conversion and image enhancement.

(*1) Grayscale Conversion*. Texture analysis is used in different machine vision problems such as surface inspection and classification. We can define texture as the spatial distribution of different gray levels in a neighborhood. To perform textural analysis it is important to convert color image into grayscale image.

(*2) Contrast Enhancement*. Image enhancement is important to improve the quality of the input image. The enhancement technique used in the proposed methodology is contrast enhancement. In proposed methodology the increase in image contrast is performed using the formula given in [25] which is based on saturating 1% of the data at high and low gray intensity values of the input image.

Contrast enhancement formula is as follows:

(1) $$\begin{array}{c} \text{CE}\left(i,j\right)=\{\begin{array}{cc} 255, & \text{if}\;f\left(i,j\right)>h,\\ 0, & \text{if}\;f\left(i,j\right)<l,\\ \min\left(\frac{f\left(i,j\right)-1}{h-l},255\right), & \text{otherwise}, \end{array} \end{array}$$

where CE (*i*, *j*) is contrast enhancement at pixel *i*, *j*, *f*(*i*, *j*) is image intensity at a particular index *i*, *j*, *h* is high intensity of the image, and *l* is low intensity of the image.

#### 2.2.2. Feature Extraction

After applying preprocessing on the input image, we need to perform feature extraction to quantify surface of the image through different parameters. Analysis of the surface of the tablets through its texture can provide great help in classifying them into correct and damaged tablets. Texture of a surface can be defined using different types of features which can be extracted from the gray level distribution of the image intensity. Statistical feature extraction methods are extensively used for the texture analysis. Different textural feature used in this study are gray level cooccurrence matrix (GLCM), histogram, run length matrix (RLM), autoregressive model (ARM), and HAAR wavelet features. So total of 281 textural features are extracted from each of the preprocessed images using MaZda (texture analysis software) designed by Szczypiński et al. [26]. The creation of *j*th dataset is shown in (2); here the value of *j* is from 1 to 9.

Formula for dataset representation is as follows:

(2) $$\begin{array}{c} {\text{dataset}}_{j}=\overset{L}{\underset{K=1}{⋃}}\text{features}\left(I_{k}\right), \end{array}$$

where  *I*_*k*_ is the *k*th input image, features (*I*_*k*_) is the feature set of the *k*th image, *L* is the total number of images for each dataset.

Detail of these features is given below.

(*1) Gray Level Cooccurrence Matrix (GLCM)*. GLCM is one of the statistical feature extraction methods which can be used to define texture of a surface. It is based on spatial relationship between pixels. Texture characterization can be performed by calculating how often pairs of pixels with specific values and in a specified spatial relationship occur in an image. MaZda provides eleven features extracted from GLCM. These are angular second moment, contrast, correlation, sum of squares, inverse difference moment, sum average, sum variance, sum entropy, entropy, difference variance, and difference entropy. In this research we have computed GLCM features for 5 between-pixel distances (1, 2, 3, 4, and 5). So total of 220 features are extracted.

(*2) Histogram Features*. Histogram features are first-order statistics based features, which are used to represent surface texture. According to Srinivasan and Shobha [27], histogram based features represent intensity concentration on all parts of the image. MaZda provides total of nine histogram features from which we have chosen four: mean (histogram's mean), variance (histogram's variance), skewness (histogram's skewness), and kurtosis (histogram's kurtosis).

(*3) Run Length Matrix (RLM)*. Run length from an input gray level image is defined by a set of consecutive, collinear pixels having same gray level. Coarseness of a texture in a specific direction can be captured using RLM [28]. MaZda provides total 20 features extracted for RLM. These features are run length nonuniformity, grey level nonuniformity, long run emphasis, short run emphasis, and fraction of image in runs. Each feature is computed in four different directions (horizontally, vertically, 45 degree, and 135 degree).

(*4) Autoregressive Model Features (ARM)*. MaZda provides 5 different features based on autoregressive model. These are theta 1 (parameter *θ*_1_), theta 2 (parameter *θ*_2_), theta 3 (parameter *θ*_3_), theta 4 (parameter *θ*_4_), and sigma (parameter *σ*).

(*5) HAAR Wavelet Features*. Wavelet energy feature is measured at 8 scales using four bands of frequency (LL, LH, HL, and HH) using MaZda. This provides total of 32 features.

#### 2.2.3. Feature Reduction

The feature extraction phase results in 281 different features which are very hard to deal with. So for better results it is important to reduce the dimensionality of the feature set. Three different feature reduction techniques are used in this research for extracting the most promising features which can lead us towards the correct classification between DT and NDT. These three techniques are chi-square (CS), gain ratio (GR), and relief-F (RF). Feature reduction is performed by extracting top 15 features out of complete feature vector for each of the above three techniques. Feature reduction was performed using WEKA developed by Hall et al. [29]. All of these feature selection algorithms are used along with Ranker search algorithm. It is observed that top 15 features extracted from both GR and RF for our dataset are the same. The top 15 features extracted from CS, GR, and RF are listed in Table 2. The detail of these feature selection techniques is discussed below.

(*1) Chi-Square*. Chi-square (CS) feature selection algorithm performs ranking of features by calculating chi-squared statistic for each class. CS calculates the degree of the dependency between attributes and a specific class. According to Chatcharaporn et al. [30], consider the formula for CS.

Formula for chi-square is as follows:

(3) $$\begin{array}{c} X^{2}=\overset{r}{\underset{i=1}{\sum }}\overset{c}{\underset{j=1}{\sum }}\frac{{\left(O_{ij}-E_{ij}\right)}^{2}}{E_{ij}}, \end{array}$$

where *O*_*ij*_ and *E*_*ij*_ is the observed and expected frequencies, respectively.

(*2) Gain Ratio*. Gain ratio (GR) ranks the attributes by compensating the bias for information gain (IG). According to Chatcharaporn et al. [30] GR can be measured by the following.

Formula for gain ratio is as follows:

(4) $$\begin{array}{c} \text{GR}=\frac{\text{IG}}{H\left(X\right)}, \end{array}$$

where *H*(*X*) is entropy of *X*. The result of the GR is always in [0, 1]. GR = 1 means that *X* can completely predict *Y*, where *Y* is the variable to be predicted, and GR = 0 indicates no relation between *X* and *Y*.

(*3) Relief-F*. Another statistical attribute selection technique used in this research is relief-F (RF). RF calculates weight for each feature using relationship between a feature and a specific class to rank it [30]. This weight calculation is based on two types of nearest neighbor probabilities. The first probability is calculated through two different classes with different feature values and the other probability of weight computation is based on the same class of two nearest neighbors with the same feature value [31].

The top 15 selected features from a total of 281 using CS, GR, and RF are given in Table 2. It can be seen from Table 2 that according to the CS among the top 15 features 14 are related to angular second moment (AngScMom) from multiple distances and one is inverse difference moment (InvDfMom) at distance 4. All 15 features extracted from CS are related to GLCM. On the other hand 14 features selected from GR are related to GLCM and one is wavelet energy from HAAR wavelet features.

#### 2.2.4. Classification

The evaluation of the features extracted from the tablet images is performed using three different types of classification algorithms, that is, SVM, KNN, and NB. In this research we have performed a comparison between the accuracies achieved from these classifiers. All experimental work for this research is performed using MATLAB. Classification is performed using all 281, the top 15 features selected using abovementioned feature reduction algorithms, and the top two from overall 281 features.

(*1) Naïve Bayes*. Naïve Bayes is a statistical learning algorithm that performs probabilistic classification based on Bayesian networks [32]. Naïve Bayes performs training by estimating prior and conditional probabilities from the dataset. Prior probability for a specific class is calculated by dividing the count of training examples falling in that class by total number of examples. On the other hand conditional probabilities are based on the frequency distribution of feature *x*_*i*_ from the training data that belong to that specific class [31]. NB is implemented using MATLAB for the experimentation. Some important studies related to drugs using naïve Bayes as a classifier are [33–36].

(*2) K-Nearest Neighbor (KNN)*. *K*-nearest neighbor (KNN) is a simple but robust algorithm that can efficiently deal with complex problems of classification. It is based on multiple parameters like how many nearest neighbors must be considered while classification and, denoted by *K*, distance of features within a dataset to determine which data belong to which group. In the proposed methodology we have implemented KNN using MATLAB with a *K* value of 2 and cosine as a distance metric.

(*3) Support Vector Machine (SVM)*. SVM uses linear equation built from the training data for partitioning the dataset. SVM works in two steps: mapping of nonlinear data from input space to feature space is performed in the first step and then similarity of the feature vectors is measured using kernel function. It can handle large feature sets with high accuracy [30]. SVM is implemented using MATLAB. Training of the datasets is performed using linear kernel function with sequential minimal optimization (SMO) method for separating hyperplanes. Hou et al. [37] have used SVM models for the recognition of SH3 domain-peptide.

## 3. Results and Discussion

In this research we have evaluated the accuracy of the proposed methodology using two different experiments. In Experiment I, leave-one-out (LOO) cross-validation method is used for the evaluation of the proposed approach. LOO cross-validation is firstly applied on each individual dataset, then on combined datasets of each environmental factor, and lastly over a combined dataset of all environmental factors. In Experiment II the accuracy of the proposed method is evaluated by using separate training and testing datasets. Each dataset is divided into two halves; so 50% of the data is used for training the proposed method and 50% of the remaining data is used for the testing of the proposed method. Classification accuracy of the proposed methodology is measured using three different types of classifiers (SVM, KNN, and NB). Feature vector is formed using a total of 281 texture based features extracted from the preprocessed images.

In Experiment I, first of all, we have used the whole 281 features as feature vector and evaluated the performance of the proposed methodology using all three classifiers based on LOO cross-validation. This classification is performed on each tablet dataset individually and then on combined datasets.  Table 3 contains the results of this experiment.

**Figure 4:**
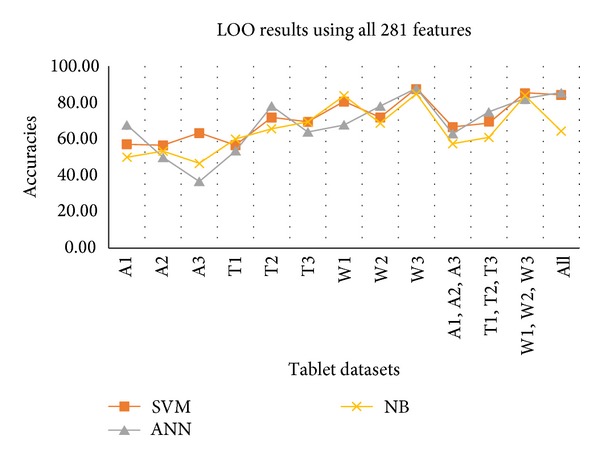
LOO results against all individual and combined datasets using 281 features.

A graphical representation of the accuracy of each classifier is shown in Figure 4. Results show that maximum accuracy is achieved by using SVM classifier for most of the datasets. Classification accuracies against moisture affected tablets are higher than the other two factors. From humidity affected tablet datasets, it can be seen that the humidity affects the surface of the solid tablets very slowly that is why they have low classification rate. Same results are reflected by the accuracies of the combined datasets.

From Table 3 to Table 8, “Acc” is for accuracy, “Sn” for sensitivity, and “Sp” for specificity.

After that LOO cross-validation is applied on the selected top 15 features. Classification accuracies are calculated again using three classifiers against the top 15 selected features and it can be seen from results that features extracted from CS provides higher accuracies compared to GR. The comparison of results using the top 15 features is shown in Table 4. Overall SVM and ANN provide higher accuracies using CS for the classification for all individual tablet datasets. SVM provides maximum 90.32% accuracy for W1 dataset using CS while ANN provides 90.91% accuracy for W3 using GR. Again from results it can be highlighted that moisture affected tablets have higher classification rate.

**Figure 5:**
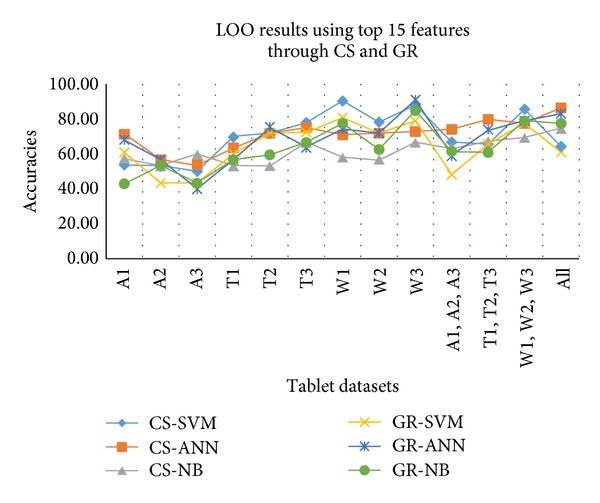
LOO results against all individual and combined datasets using top 15 features.

In case of combined datasets of tablets, the maximum accuracies achieved for moisture affected tablets and the lowest against humidity affected. In case of whole combined datasets maximum 86.30% accuracy was achieved using ANN classifier.  Figure 5 shows the accuracies of individual and combined tablet datasets.

At the end of Experiment I, we have evaluated the accuracy of the proposed method against the top two features selected from 281 features. These two features are selected by making combinations of two from 281 features and then selecting a pair of features providing maximum accuracy. The top two selected features are “S (5, 0) Entropy” (entropy at distance 5) and “Horzl_GLevNonU” (horizontal gray level nonuniformity). Entropy measure is from GLCM and Horzl_GlevNonU is from RLM.

**Figure 6:**
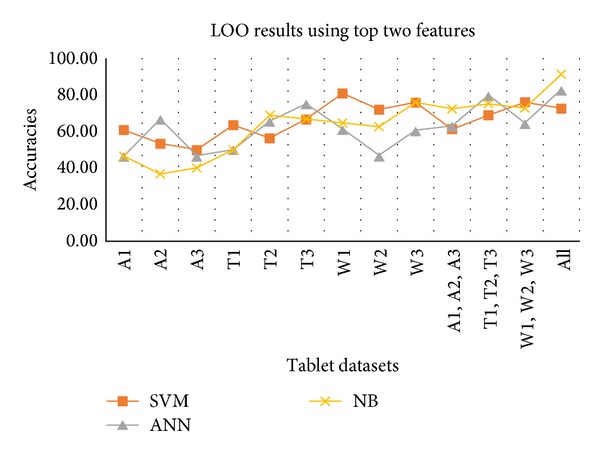
LOO results against all individual and combined datasets using top 2 features.

Table 5 shows the accuracies of individual and combined datasets. LOO cross-validation using the top two features again provides maximum classification rates for moisture affected datasets through SVM. In case of combined dataset NB provides maximum classification accuracy, that is, 91.10%, but with low sensitivity rate, that is, 29.41%. This is depicted in Figure 6.

Similarly, in Experiment II, we have evaluated the accuracies of the proposed methodology through train and test model against all 281, selected top 15, and the top two features. All accuracies in this experiment are calculated by providing the test datasets to a trained model.

**Figure 7:**
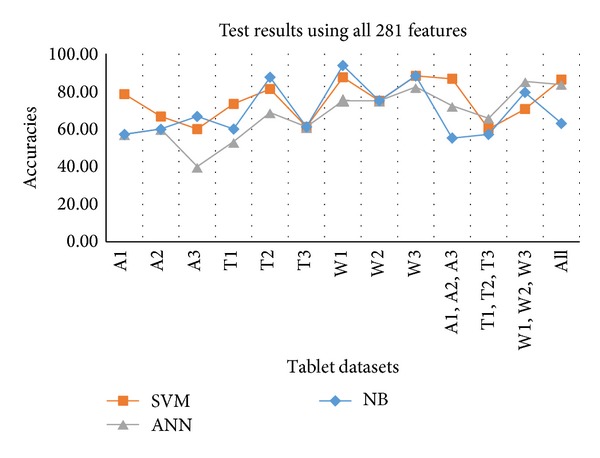
Test results against all individual and combined datasets using all 281 features.

Table 6 shows the test results against all 281 features. In case of overall combined dataset, 86.30% accuracy and for combined humidity dataset 86.67% accuracy were achieved through SVM. Against individual datasets like temperature and moisture NB provides more accurate results. NB provides 93.75% accuracy for W1 (with 100% sensitivity and 85.71% specificity) and provides for T2 87.50%. In case of A1, SVM provides maximum accuracy, that is, 78.57%.  Figure 7 shows the results in graphical form.

**Figure 8:**
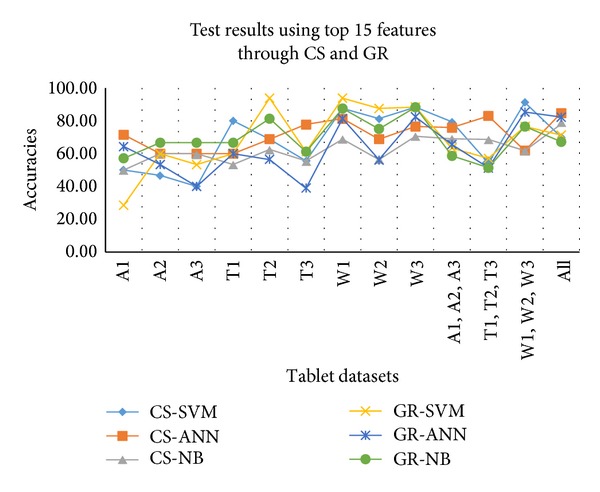
Test results against all individual and combined datasets using top 15 features.

The test results against the selected top 15 features are shown in Table 7. The features selected from CS outperform than GR in most of the cases. NB provides relatively low accuracies than SVM and ANN. In case of tablets affected by humidity and temperature ANN provides better accuracies but, for moisture affected tablets, SVM is better. When the trained model is tested on combined datasets, maximum 91.18% accuracy is achieved against moisture affected tablets datasets. The graphical representation of these results is shown in Figure 8.

**Figure 9:**
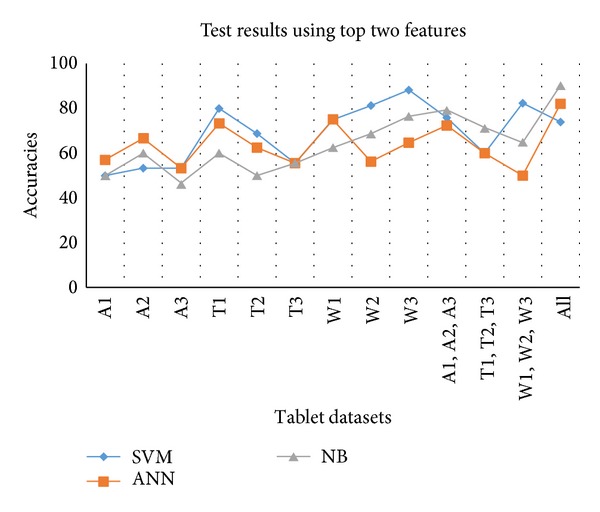
Test results against all individual and combined datasets using top 2 features.

The accuracies against the top two selected features using test datasets are provided in Table 8. It can be seen from results that for almost all of the datasets SVM is better except for humidity. In case of humidity affected datasets ANN provides better results. For W3, SVM provides 88.24% accuracy with 88.89% sensitivity and 87.5% specificity. In case of overall combined dataset NB provides maximum accuracy, that is, 90.14%, with 98.48% specificity. These results are also shown in Figure 9.

## 4. Conclusion

In this research we have proposed a new methodology for the classification of defective and nondefective tablets using image processing and machine learning techniques. In proposed approach we have used textural features extracted from the surface of the preprocessed images. Whole analysis is performed on nondefective and defective tablets. The surfaces of defective tablets are affected by three environmental factors, that is, temperature, humidity, and moisture. Comparison analysis is performed using all 281, the top 15 (extracted using CS, GR, and RF), and the top 2 features. Classification is performed using SVM, KNN, and NB classifiers. Analysis shows that higher accuracies are achieved on moisture affected tablets as moisture has quick reaction with the APIs of the tablet. In different types of experiments, the proposed methodology using SVM for most of the datasets is better than the other two classifiers. In future the combination of spatial and spectral data of the tablets can be used to achieve higher accuracies.

## Figures and Tables

**Table 1:** Dataset description.

Environmental factors	Dataset	Number of DT	Number of NDT
Humidity	A1	11	17
	A2	13	17
	A3	13	17

Temperature	T1	13	17
	T2	15	17
	T3	19	17

Moisture	W1	14	17
	W2	15	17
	W3	16	17

**Table 2:** List of top 15 selected features from CS, GR, and RF.

Rank	Features	
	Chi-square	Gain ratio/relief-F
1	S(5, 5)AngScMom	WavEnHH_s-8
2	S(4, −4)AngScMom	S(3, 0)SumOfSqs
3	S(0, 4)AngScMom	S(3, 0)Contrast
4	S(0, 2)AngScMom	S(3, 0)AngScMom
5	S(4, 4)AngScMom	S(2, −2)DifEntrp
6	S(2, 2)AngScMom	S(2, −2)DifVarnc
7	S(4, 0)AngScMom	S(2, −2)Entropy
8	S(2, −2)AngScMom	S(3, 0)Correlat
9	S(0, 3)AngScMom	S(3, 0)InvDfMom
10	S(3, −3)AngScMom	S(2, −2)SumVarnc
11	S(3, 0)AngScMom	S(3, 0)SumAverg
12	S(4, 4)InvDfMom	S(3, 0)DifEntrp
13	S(2, 0)AngScMom	S(3, 0)DifVarnc
14	S(3, 3)AngScMom	S(3, 0)Entropy
15	S(1, −1)AngScMom	S(3, 0)SumEntrp

**Table 3:** LOO results for all individual and combined datasets using 281 features.

Datasets	Number of features	SVM			KNN			NB		
		Acc	Sn	Sp	Acc	Sn	Sp	Acc	Sn	Sp
A1	281	57.14	64.71	45.45	67.86	64.71	72.73	50.00	47.06	54.55
A2	281	56.67	58.82	53.85	50.00	41.18	61.54	53.33	47.06	61.54
A3	281	63.33	64.71	61.54	36.67	29.41	46.15	46.67	35.29	61.54
T1	281	56.67	58.82	53.85	53.33	58.82	46.15	60.00	47.06	76.92
T2	281	71.88	70.59	73.33	78.13	82.35	73.33	65.63	64.71	66.67
T3	281	69.44	70.59	68.42	63.89	58.82	68.42	69.44	88.24	52.63
W1	281	80.65	82.35	78.57	67.74	58.82	78.57	83.87	82.35	85.71
W2	281	71.88	76.47	66.67	78.13	76.47	80	68.75	64.71	73.33
W3	281	87.88	82.35	93.75	87.88	88.24	87.5	84.85	94.12	75
A1, A2, and A3	281	66.67	52.94	72.97	62.96	17.65	83.78	57.41	35.29	67.57
T1, T2, and T3	281	68.75	58.82	72.34	75.00	41.18	87.23	60.94	70.59	57.45
W1, W2, and W3	281	85.48	70.59	91.11	82.26	58.82	91.11	83.87	76.47	86.67
All	281	84.25	52.94	88.37	85.62	11.76	95.35	64.38	58.82	65.12

**Table 4:** LOO results for all individual and combined datasets using top 15 features.

Datasets	Number of features	FR-algorithm	SVM			KNN			NB		
			Acc	Sn	Sp	Acc	Sn	Sp	Acc	Sn	Sp
A1	15	CS	53.57	47.06	63.64	71.43	82.35	54.55	57.14	41.18	81.82
A2	15	CS	53.33	47.06	61.54	56.67	58.82	53.85	53.33	41.18	69.23
A3	15	CS	50.00	47.06	53.85	53.33	64.71	38.46	60.00	47.06	76.92
T1	15	CS	70.00	82.35	53.85	63.33	70.59	53.85	53.33	35.29	76.92
T2	15	CS	71.88	94.12	46.67	71.88	76.47	66.67	53.13	41.18	66.67
T3	15	CS	77.78	94.12	63.16	75.00	70.59	78.95	66.67	52.94	78.95
W1	15	CS	90.32	88.24	92.86	70.97	76.47	64.29	58.06	52.94	64.29
W2	15	CS	78.13	94.12	60	71.88	76.47	66.67	56.25	35.29	80
W3	15	CS	87.88	94.12	81.25	72.73	70.59	75	66.67	47.06	87.5
A1, A2, and A3	15	CS	66.67	41.18	78.38	74.07	52.94	83.78	62.96	35.29	75.68
T1, T2, and T3	15	CS	65.63	94.12	55.32	79.69	52.94	89.36	67.19	41.18	76.6
W1, W2, and W3	15	CS	85.48	94.12	82.22	77.42	58.82	84.44	69.35	29.41	84.44
All	15	CS	64.38	94.12	60.47	86.30	35.29	93.02	74.66	29.41	80.62

A1	15	GR	60.71	64.71	54.55	67.86	76.47	54.55	42.86	47.06	36.36
A2	15	GR	43.33	47.06	38.46	56.67	70.59	38.46	53.33	47.06	61.54
A3	15	GR	43.33	35.29	53.85	40.00	47.06	30.77	43.33	35.29	53.85
T1	15	GR	60.00	58.82	61.54	56.67	58.82	53.85	56.67	47.06	69.23
T2	15	GR	71.88	70.59	73.33	75.00	82.35	66.67	59.38	52.94	66.67
T3	15	GR	72.22	88.24	57.89	63.89	64.71	63.16	66.67	82.35	52.63
W1	15	GR	80.65	76.47	85.71	74.19	76.47	71.43	77.42	76.47	78.57
W2	15	GR	71.88	70.59	73.33	71.88	70.59	73.33	62.50	64.71	60
W3	15	GR	78.79	88.24	68.75	90.91	94.12	87.5	84.85	94.12	75
A1, A2, and A3	15	GR	48.15	35.29	54.05	59.26	35.29	70.27	61.11	35.29	72.97
T1, T2, and T3	15	GR	65.63	70.59	63.83	73.44	47.06	82.98	60.94	47.06	65.96
W1, W2, and W3	15	GR	77.42	70.59	80	79.03	64.71	84.44	79.03	64.71	84.44
All	15	GR	60.96	64.71	60.47	82.88	23.53	90.7	77.40	47.06	81.4

**Table 5:** LOO results for all individual and combined datasets using top 2 features.

Datasets	Number of features	Feature name	SVM			KNN			NB		
			Acc	Sn	Sp	Acc	Sn	Sp	Acc	Sn	Sp
A1	2	A189 and A226	60.71	41.18	90.91	46.43	58.82	27.27	46.43	47.06	45.45
A2	2	A189 and A226	53.33	47.06	61.54	66.67	76.47	53.85	36.67	41.18	30.77
A3	2	A189 and A226	50.00	47.06	53.85	46.67	52.94	38.46	40.00	47.06	30.77
T1	2	A189 and A226	63.33	52.94	76.92	50.00	52.94	46.15	50.00	47.06	53.85
T2	2	A189 and A226	56.25	64.71	46.67	65.63	64.71	66.67	68.75	58.82	80
T3	2	A189 and A226	66.67	58.82	73.68	75.00	70.59	78.95	66.67	64.71	68.42
W1	2	A189 and A226	80.65	70.59	92.86	61.29	70.59	50	64.52	58.82	71.43
W2	2	A189 and A226	71.88	76.47	66.67	46.88	52.94	40	62.50	58.82	66.67
W3	2	A189 and A226	75.76	76.47	75	60.61	58.82	62.5	75.76	64.71	87.5
A1, A2, and A3	2	A189 and A226	61.11	35.29	72.97	62.96	35.29	75.68	72.22	29.41	91.89
T1, T2, and T3	2	A189 and A226	68.75	52.94	74.47	79.69	47.06	91.49	75.00	35.29	89.36
W1, W2, and W3	2	A189 and A226	75.81	70.59	77.78	64.52	41.18	73.33	72.58	29.41	88.89
All	2	A189 and A226	72.60	70.59	72.87	82.88	23.53	90.7	91.10	29.41	99.22

**Table 6:** Accuracies for test datasets using 281 features.

Datasets	Number of features	SVM			KNN			NB		
		Acc	Sn	Sp	Acc	Sn	Sp	Acc	Sn	Sp
A1	281	78.57	80	42.86	57.14	60	28.57	57.14	40	57.14
A2	281	66.67	55.56	83.33	60.00	66.67	50	60.00	66.67	50
A3	281	60.00	66.67	50	40.00	33.33	50	66.67	77.78	50
T1	281	73.33	66.67	83.33	53.33	44.44	66.67	60.00	55.56	66.67
T2	281	81.25	77.78	85.71	68.75	66.67	71.43	87.50	77.78	100
T3	281	61.11	75	50	61.11	62.5	60	61.11	75	50
W1	281	87.50	88.89	85.71	75.00	55.56	100	93.75	100	85.71
W2	281	75.00	77.78	71.43	75.00	66.67	85.71	75.00	77.78	71.43
W3	281	88.24	100	75	82.35	77.78	87.5	88.24	100	75
A1, A2, and A3	281	86.67	57.14	100	72.41	42.86	81.82	55.17	28.57	63.64
T1, T2, and T3	281	60.00	57.14	60.71	65.71	57.14	67.86	57.14	57.14	57.14
W1, W2, and W3	281	70.59	42.86	77.78	85.29	42.86	96.3	79.41	14.29	96.3
All	281	86.30	28.57	92.42	83.56	14.29	90.91	63.01	57.14	63.64

**Table 7:** Accuracies for test datasets using top 15 features.

Datasets	Number of features	FR-algorithm	SVM			KNN			NB		
			Acc	Sn	Sp	Acc	Sn	Sp	Acc	Sn	Sp
A1	15	CS	50.00	30	57.14	71.43	80	28.57	50.00	30	57.14
A2	15	CS	46.67	33.33	66.67	60.00	44.44	83.33	60.00	44.44	83.33
A3	15	CS	40.00	33	50	60.00	67	50	60.00	44	83.33
T1	15	CS	80.00	66.67	100	60.00	66.67	50	53.33	33.33	83.33
T2	15	CS	68.75	44.44	100	68.75	77.78	57.14	62.50	44.44	85.71
T3	15	CS	55.56	75	40	77.78	87.5	70	55.56	37.5	70
W1	15	CS	87.50	100	71.43	81.25	77.78	85.71	68.75	77.78	57.14
W2	15	CS	81.25	100	57.14	68.75	66.67	71.43	56.25	44.44	71.43
W3	15	CS	88.24	100	75	76.47	77.78	75	70.59	44.44	100
A1, A2, and A3	15	CS	79.31	14.29	100	75.86	42.86	86.36	68.97	14.29	86.36
T1, T2, and T3	15	CS	54.29	85.71	46.43	82.86	71.43	85.71	68.57	14.29	82.14
W1, W2, and W3	15	CS	91.18	85.71	92.59	61.76	42.86	66.67	61.76	28.57	70.37
All	15	CS	69.86	85.71	68.18	84.93	14.29	92.42	78.08	14.29	84.85

A1	15	GR	28.57	20	28.57	64.29	80	14.29	57.14	40	57.14
A2	15	GR	60.00	55.56	66.67	53.33	66.67	33.33	66.67	66.67	66.67
A3	15	GR	53.33	67	33.33	40.00	33	50	66.67	78	50
T1	15	GR	60.00	66.67	50	60.00	44.44	83.33	66.67	66.67	66.67
T2	15	GR	93.75	88.89	100	56.25	44.44	71.43	81.25	66.67	100
T3	15	GR	61.11	87.5	40	38.89	50	30	61.11	75	50
W1	15	GR	93.75	100	85.71	81.25	88.89	71.43	87.50	88.89	85.71
W2	15	GR	87.50	88.89	85.71	56.25	55.56	57.14	75.00	77.78	71.43
W3	15	GR	88.24	100	75	82.35	88.89	75	88.24	100	75
A1, A2, and A3	15	GR	63.33	28.57	77.27	65.52	57.14	68.18	58.62	14.29	72.73
T1, T2, and T3	15	GR	57.14	71.43	53.57	51.43	85.71	42.86	51.43	57.14	50
W1, W2, and W3	15	GR	76.47	42.86	85.19	85.29	57.14	92.59	76.47	14.29	92.59
All	15	GR	71.23	14.29	77.27	82.19	28.57	87.88	67.12	14.29	72.73

**Table 8:** Accuracies for test datasets using top 2 features.

Datasets	Number of features	Feature name	SVM			KNN			NB		
			Acc	Sn	Sp	Acc	Sn	Sp	Acc	Sn	Sp
A1	2	A189 and A226	50	30	57.14	57.14	70	14.29	50	50	28.57
A2	2	A189 and A226	53.33	55.56	50	66.67	66.67	66.67	60	55.56	66.67
A3	2	A189 and A226	53.33	55.56	50	53.33	66.67	33.33	46.67	33.33	66.67
T1	2	A189 and A226	80	77.78	83.33	73.33	88.89	50	60	44.44	83.33
T2	2	A189 and A226	68.75	44.44	100	62.5	55.56	71.43	50	44.44	57.14
T3	2	A189 and A226	55.56	75	40	55.56	75	40	55.56	62.5	50
W1	2	A189 and A226	75	66.67	85.71	75	100	42.86	62.5	66.67	57.14
W2	2	A189 and A226	81.25	88.89	71.43	56.25	66.67	42.86	68.75	66.67	71.43
W3	2	A189 and A226	88.24	88.89	87.5	64.71	55.56	75	76.47	55.56	100
A1, A2, and A3	2	A189 and A226	75.86	14.29	95.45	72.41	42.86	81.82	79.31	14.29	100
T1, T2, and T3	2	A189 and A226	60	71.43	57.14	60	42.86	64.29	71.43	14.29	85.71
W1, W2, and W3	2	A189 and A226	82.35	57.14	88.89	50	42.86	51.85	64.71	28.57	74.07
All	2	A189 and A226	73.97	57.14	75.76	82.19	28.57	87.88	90.41	14.29	98.48
